# Synergistic Control of *Blattella germanica* by Beta-Cypermethrin and *Metarhizium anisopliae*: Disruption of Gut Microbiota, Histopathology, and Detoxification Systems

**DOI:** 10.3390/insects17090896

**Published:** 2026-08-26

**Authors:** Xiaoyan Wu, Yiwen Wang, Tong Cai, Caixia Liu, Rong Huang, Jianzheng Wang, Xuejun Wang, Fan Zhang

**Affiliations:** 1 Medical School, Shandong Xiehe University, Jinan 250109, China; 2 Shandong Province Key Laboratory of Emerging Contaminants Risk Prevention and Control, College of Life Science, Shandong Normal University, Jinan 250358, China; 3 Dongying Key Laboratory of Salt Tolerance Mechanism and Application of Halophytes, Dongying Institute, Shandong Normal University, Dongying 257000, China; 4 Shandong Center for Disease Control and Prevention, Jinan 250013, China; 5 Key Laboratory of Animal Resistance Biology of Shandong Province, College of Life Science, Shandong Normal University, 88 East Wenhua Road, Jinan 250014, China

**Keywords:** *Metarhizium anisopliae*, beta-cypermethrin, synergistic effect, gut microbiota, detoxification enzymes

## Abstract

The *Blattella germanica* L. (Blattodea: Ectobiidae) is a major urban pest with widespread insecticide resistance. This study evaluated the synergistic effect of combining *Metarhizium anisopliae* with beta-cypermethrin (β-CYP) and investigated its mechanisms. Compatibility assays showed that β-CYP (1–10 μg/mL) was highly compatible with *M. anisopliae* (1 × 10^7^–1 × 10^9^ cfu/mL), with enhanced compatibility at lower concentrations. Bioassays confirmed significant synergy, increasing mortality and shortening median lethal time. Mechanistically, the combination accelerated histopathological damage to the midgut, hindgut, Malpighian tubules, and fat body, while promoting fungal proliferation in hemolymph and gut tissues. 16S rRNA sequencing revealed gut microbiota dysbiosis: Firmicutes replaced Bacteroidetes as the dominant phylum, opportunistic pathogens (*Weissella*, *Alistipes*) increased, and beneficial bacteria (*Enterococcus*, *Fusobacterium*) decreased. *Weissella* recolonization experiments confirmed its mortality-enhancing role. Additionally, the combination suppressed carboxylesterase activity and modulated immune gene expression (*CYP4G19*, *BgPo*), compromising host defense. Collectively, these findings demonstrate that β-CYP and *M. anisopliae* exert synergistic effects through multi-target mechanisms, offering a promising integrated pest management strategy that reduces chemical usage while enhancing biocontrol efficacy.

## Simple Summary

The German cockroach, the most common indoor cockroach pest across the world, has developed strong resistance to many widely used chemical insecticides, while eco-friendly fungal pest control agents work too slowly when used alone to deliver fast, practical control. This study tested whether combining a very low dose of the common household insecticide beta-cypermethrin with the insect-killing fungus *Metarhizium anisopliae* could improve control efficacy and investigated how the combination works. We found the two agents worked far better together than either used alone: the combination killed cockroaches much faster by suppressing the enzymes cockroaches use to break down insecticides, disrupting the balance of beneficial microbes in their gut, and damaging key internal tissues. This strategy reduces chemical insecticide usage, slows the development of pesticide resistance, and provides a safer, more environmentally friendly option for cockroach management in homes and public areas.

## 1. Introduction

The German cockroach (*Blattella germanica* L.) is one of the most important indoor pests worldwide due to its rapid reproduction and close association with human habitats. In addition to mechanical transmission of pathogens, its secretions and carcasses contain potent allergens that can trigger asthma and other allergic reactions, posing a significant public health threat [1,2].

Chemical control remains the primary method for cockroach management, but the widespread use of this method has led to strong resistance in cockroaches and negative impacts on the ecological environment [3,4]. Pyrethroid insecticides (including beta-cypermethrin, β-CYP) are widely used due to their high efficacy, low mammalian toxicity, and low environmental persistence, accounting for approximately 25% of the global insecticide market [5,6]. However, long-term improper use has led to widespread insecticide resistance in *B. germanica* populations [7]. Resistance mechanisms are complex, with enhanced metabolic detoxification mediated by cytochrome P450s, esterases, or glutathione S-transferases being a common mechanism in German cockroaches [8,9]; in addition, reduced cuticular penetration is also a key factor, as demonstrated by the much higher toxicity of injected versus topically applied pyrethroids, indicating that the cuticle serves as an important barrier to insecticide entry [10].

Because of the increasing challenge of insecticide resistance, current cockroach management increasingly relies on integrated approaches that combine chemical control with sanitation, resistance management, and alternative control measures. Gel baits remain an effective component of integrated cockroach management, particularly when combined with sanitation and other control measures [11,12]. Previous studies have demonstrated substantial population suppression using various gel bait formulations against German cockroaches [13], although their performance may vary among products, cockroach populations, and environmental conditions [14,15]. While chemical synergists can enhance the efficacy of certain insecticides, particularly pyrethroids, their effectiveness can vary depending on the insecticide, resistance mechanism, and cockroach population. Thus, chemical synergists alone are unlikely to provide a broadly applicable strategy for improving control efficacy. Instead, integrated strategies incorporating control agents with different modes of action may provide an alternative approach to improving control efficacy while reducing reliance on single-mode chemical treatments.

Entomopathogenic fungi, particularly *M. anisopliae*, have attracted considerable attention as potential biological control agents against German cockroaches [16,17]. However, despite considerable research and development of entomopathogenic fungi as biological control agents, their application in practical German cockroach management remains limited [18,19,20]. Although entomopathogenic fungi offer advantages such as safety and a relatively low risk of resistance development, their slow action and variable efficacy under field conditions may limit their practical application. *M. anisopliae* penetrates the host cuticle by forming germ tubes and producing chitinase, enters the hemocoel, secretes destruxins and other toxins, depletes host nutrients, obstructs hemolymph circulation, destroys vital tissues, and ultimately causes cockroach death [21,22,23,24,25]. In this context, combining entomopathogenic fungi with conventional insecticides represents a potentially synergistic biological–chemical control strategy that integrates distinct modes of action and may enhance control efficacy while reducing reliance on single-mode chemical treatments.

Numerous studies have shown that *M. anisopliae* has good compatibility with various chemical insecticides, and the combination of these two agents has achieved significant progress in the field of pest control. For example, sublethal doses of imidacloprid combined with *M. anisopliae* significantly reduced the survival rate of *Aedes aegypti*, with better efficacy than the fungus alone [26]; the combination of *Beauveria bassiana* with low concentrations of nitenpyram significantly increased the mortality of all stages of the red palm weevil, showing significant synergistic effects [27]. Additionally, sublethal doses of beta-cypermethrin combined with *Metarhizium rileyi* enhanced virulence against *Spodoptera litura* [28]. More notably, the transgenic *Metarhizium pinghaense* expressing spider neurotoxin (Met-Hybrid) showed a strong synergistic effect with pyrethroid (permethrin) in resistant *Anopheles* mosquitoes: five days after infection with Met-Hybrid, 89% of resistant mosquitoes exposed to permethrin died within 24 h, while only 22% of mosquitoes infected with Met-Hybrid alone died during the same period. Combining *M. anisopliae* with chlorantraniliprole against locusts and measuring changes in 10 enzyme activities revealed that the synergistic effect led to increased locust mortality [29]. These studies fully demonstrate the great potential of combining *M. anisopliae* with pyrethroid insecticides for resistant pest management.

However, studies on insecticide–fungus combinations against urban pests such as cockroaches remain very limited, and the comprehensive mechanisms underlying such synergy—particularly the dynamics of gut microbiota, which are critically important for insect health and pathogen defense—have not been systematically investigated [30]. Existing studies have investigated the combinations of *Metarhizium* with hydramethylnon and *Metarhizium* with boric acid [31,32]. The present study seeks to further expand the research horizon beyond these previous investigations. Therefore, this study evaluated the synergistic effect of β-CYP combined with *M. anisopliae* against *B. germanica* and explored the underlying mechanisms through histopathology, fungal proliferation quantification, gut microbiota analysis (16S sequencing), detoxification enzyme activity assays (GST, CarE), and immune gene expression (*CYP4G19*, *BgPo*), with functional validation of key bacterial taxa via recolonization experiments. These findings provide a theoretical foundation for developing integrated pest management strategies that reduce synthetic insecticide usage while enhancing biocontrol efficacy.

## 2. Materials and Methods

### 2.1. Insects

The German cockroaches used in this study were adult males of the susceptible laboratory strain (SD strain). This strain was originally collected from a residential area in Jinan, Shandong Province, China, in 1998 and has been continuously reared in the Shandong Provincial Key Laboratory of Animal Resistance Biology for more than 15 years without exposure to any chemical insecticides. Its susceptibility to common insecticides (including β-CYP, imidacloprid, and fipronil) is routinely verified in our laboratory every 6 months via topical bioassays, and the LC_50_ values have remained stable for the past 10 years, confirming its stable susceptible phenotype. The colony is maintained in large populations (no less than 500 breeding adults per generation, with random mating between generations) following standard laboratory rearing protocols for *B. germanica* to minimize the effect of genetic drift. The cockroaches were fed with rodent diet (Keao Xieli (Tianjin) Co., Ltd., Tianjin, China) and provided with tap water ad libitum. They were kept under rearing conditions of 26 ± 1 °C, with a photoperiod of 12 h light: 12 h dark and a relative humidity of 60 ± 5%.

### 2.2. Pesticides, Fungi and Bacteria

The *M. anisopliae* strain EB0732 was isolated and purified by the Shandong Provincial Key Laboratory of Animal Resistance Biology [33]. The strain, previously stored at −80 °C, was revived and then inoculated onto PDA solid medium using the streak plate method. The Petri dishes were sealed with sealing film and incubated upside down in a microbiological incubator at 28 ± 1 °C for 14 days. The cultured plates were subsequently stored at 4 °C for future use.

The *Weissella* sp. strain used in this study was isolated, identified, and preserved in our laboratory from the gut of healthy German cockroaches [34]. Prior to the experiment, the strain was inoculated into MRS medium and incubated at 28 °C with shaking until the logarithmic growth phase. Saline solution consisted of 0.9% NaCl (*w*/*v*) with a molarity of 0.154 M. The bacterial cells were collected by centrifugation, resuspended in sterile saline, and adjusted to a concentration of 1 × 10^9^ cfu/mL for subsequent use.

### 2.3. Compatibility of β-CYP with M. anisopliae

To evaluate the compatibility of β-CYP with *M. anisopliae* before combined application, we measured spore germination, mycelial growth, and sporulation. Under sterile conditions, *M. anisopliae* spore suspensions at a concentration of 1 × 10^8^ cfu/mL were prepared in germination broth and mixed with different volumes of acetone to obtain final acetone concentrations of 0% (control), 0.5%, 1%, 5%, and 10%. An aliquot of 5 μL of each mixture was spread onto glass slides, placed in humidified Petri dishes, and incubated at 28 ± 1 °C for 24 h. Spore germination was observed in five randomly selected fields of view per slide (germination defined as germ tube length ≥ half the spore diameter). Each treatment was repeated three times, and the spore germination inhibition rate was calculated. Simultaneously, acetone was added to PDA medium before solidification to prepare plates with corresponding acetone concentrations. A 5 μL aliquot of the 1 × 10^8^ cfu/mL *M. anisopliae* suspension was inoculated onto the center of each plate, followed by incubation at 28 ± 1 °C. The colony diameter was measured every five days using the cross method. After 15 days of incubation, the colony diameter inhibition rate was calculated. Additionally, after 15 days of incubation, spores were scraped from the plate surface, suspended in 1 mL of sterile water containing 0.1% Tween-80, and vortexed thoroughly. The spore concentration was determined using a hemocytometer, and the sporulation inhibition rate was calculated. Based on acetone compatibility results, 1% acetone was selected as the solvent for subsequent experiments. Compatibility of β-CYP with *M. anisopliae* was determined using the same methods, with β-CYP solutions at 1, 5, and 10 μg/mL (containing 1% acetone) mixed with *M. anisopliae* suspensions at 1 × 10^7^, 1 × 10^8^, and 1 × 10^9^ cfu/mL.

The compatibility was evaluated using the biological index (BI) proposed by Rossi-Zalaf et al. [35]:

BI = [VG × SP × GER]/100^2^ (1)

where VG represents the percentage of vegetative growth relative to the control group, SP represents the percentage of sporulation relative to the control group, and GER represents the percentage of conidial germination relative to the control group. BI values from 0 to 41 are classified as toxic, 42 to 66 as moderately toxic, and greater than 66 as compatible.

### 2.4. Virulence Bioassays

Healthy male adult German cockroaches were selected for virulence assays. The following treatment groups were established: β-CYP alone (1, 3, 5, and 7 μg/mL, dissolved in 1% acetone); *M. anisopliae* alone (1 × 10^7^, 1 × 10^8^, and 1 × 10^9^ cfu/mL, prepared in 1% acetone); combination treatment consisting of β-CYP and *M. anisopliae* at the corresponding concentrations, prepared in 1% acetone and applied topically as a total volume of 2 μL per insect; and a solvent control consisting of 2 μL of 1% acetone. Thus, the solvent control was matched to the combination treatment with respect to application volume and acetone concentration. Each group consisted of 20 insects, and three independent biological replicates were performed. Following treatment, mortality was recorded daily for 15 days.

The synergistic effect was evaluated using Mansour’s co-toxicity factor method [36]: Co-toxicity factor = (observed mortality – expected mortality)/expected mortality × 100, where expected mortality = mortality of β-CYP alone + mortality of *M. anisopliae* alone × (1 − mortality of β-CYP alone). Factors > 20 indicate synergism, <−20 indicate antagonism, and values between −20 and 20 indicate additive effects.

Probit regression analysis was performed to determine the median lethal time (LT_50_) with 95% confidence intervals.

### 2.5. Histopathological Examination

Based on bioassay results, the combination of 1 μg/mL β-CYP and 1 × 10^7^ cfu/mL *M. anisopliae* (which showed a significant synergistic effect according to the co-toxicity factor analysis) was selected for histopathological examination. Cockroaches were treated with β-CYP alone, *M. anisopliae* alone, or the combination. After 6 days, insect abdomens were dissected and fixed in Carnoy’s solution for 24 h. After decalcification, samples were rinsed with sterile PBS, washed thoroughly with 95% ethanol, and subjected to gradient dehydration using ethanol series (75%, 85%, 90%, 95%, and 100%). Samples were then cleared with xylene and embedded in paraffin at 60 °C. Sections (5 μm) were deparaffinized, stained with hematoxylin and eosin, and mounted [37]. Images were observed and captured using CaseViewer2.4 software.

### 2.6. Quantification of M. anisopliae Relative Abundance

Hemolymph and gut samples were collected at 2, 4, and 6 days post-treatment from 15 healthy adult male German cockroaches in each group (*M. anisopliae* alone, combination, and control). DNA was extracted using a plant/fungal DNA kit (SiMGEN) with modifications: prior to proteinase K addition, 20 μL of lysozyme (20 mg/mL) was added and incubated at 37 °C for 1 h.

Quantitative real-time PCR (qRT-PCR) was performed to quantify the relative abundance of *M. anisopliae* in the samples. For each biological sample, qRT-PCR assays were conducted in triplicate technical replicates to ensure measurement precision. For each time point, 5 cockroaches were sacrificed and pooled as one biological replicate. A total of 3 independent biological replicates were set up for each treatment group, and each biological replicate was subjected to 3 technical replicates to ensure reliable experimental results and sufficient DNA yield. The 2^−ΔΔCT^ method was used to calculate relative expression levels [38].

### 2.7. Gut Microbiota Analysis

A total of 480 healthy adult male German cockroaches were divided into four groups: control, *M. anisopliae* alone, β-CYP alone, and combination. Using a micropipette, 2 μL of concentration of β-CYP solution or *M. anisopliae* suspension was applied topically to the abdomen of German cockroaches. The control group received 2 μL of 1% acetone, matching the solvent and application volume used in the combination treatment. Gut samples were collected on day 4 post-treatment (three biological replicates per group). Total genomic DNA was extracted from gut microorganisms using a DNA extraction kit (Tiangen Biotech Co., Ltd., Beijing, China). The V3–V4 hypervariable regions of the 16S rRNA gene were amplified using primers 338F and 806R. PCR products were sequenced on an Illumina platform. Raw sequences were processed using FLASH and Trimmagic. Operational taxonomic units (OTUs) were clustered at 97% similarity threshold. Alpha diversity indices were analyzed using Mothur software. Multiple algorithms were used to present the beta diversity matrix of species. Principal Coordinates Analysis (PCoA) and Non-metric Multidimensional Scaling (NMDS) analyses were plotted based on the R language platform. Raw sequencing data were added to the NCBI Short Read Archive (SRA) BioProject PRJNA1452616.

### 2.8. Weissella Recolonization Experiment

Based on the sequencing results showing increased *Weissella* abundance in the combination group, recolonization experiments were performed. Healthy adult male German cockroaches were divided into three groups: an oral-recolonization group, a hemocoel-injection group, and a non-recolonized control group. Each group consisted of three independent biological replicates, with 20 cockroaches per replicate (*n* = 60 per group). To deplete the gut microbiota, cockroaches were fed for 4 days with sterile cotton soaked in an antibiotic solution containing 62 μg/mL levofloxacin and 62 μg/mL gentamicin, together with sterile rodent diet (Keao Xieli (Tianjin) Co., Ltd., Tianjin, China); the cotton and diet were replaced daily. Following microbiota depletion, *Weissella* was reintroduced by oral administration or hemocoel injection of 2 μL of *Weissella* suspension (1 × 10^9^ cfu/mL). Two days after *Weissella* colonization in vivo, the combination treatment (1 μg/mL β-CYP and 1 × 10^9^ cfu/mL *M. anisopliae* in 1% acetone) was applied topically at 2 μL per insect. The non-recolonized control group received the same combination treatment without *Weissella* recolonization. Mortality was recorded daily.

### 2.9. Detoxification Enzyme Assays

Healthy male adults were randomly divided into four groups: control, β-CYP alone, *M. anisopliae* alone, and combination. Samples were collected at 1, 2, 3, 4, and 5 days post-treatment, with nine cockroaches per group at each time point, pooled into three independent biological replicates (three individuals per replicate).

Glutathione S-transferase (GST) activity was quantified using a commercial colorimetric kit (Beijing Solarbio Biotechnology Co., Ltd., Beijing, China; Cat# BC0355) with 1-chloro-2,4-dinitrobenzene (CDNB) as the substrate. Midgut tissues were homogenized in ice-cold Reagent I using Low-temperature grinder (SWE-FP, Servicebio, Wuhan, China) with all operations performed on ice. The homogenate was then centrifuged at 8000× *g* for 10 min at 4 °C using a high-speed refrigerated centrifuge (Eppendorf 5810R, Eppendorf AG, Hamburg, Germany), and the resulting supernatant was collected for measurement. The reaction was initiated by adding the supernatant to a mixture of reagent II and reagent III, and the absorbance at 340 nm was recorded using a SpectraMax M5 microplate reader (Molecular Devices, Sunnyvale, CA, USA) at 10 s and after 5 min incubation at 37 °C. GST activity was calculated as U/mg protein, where one unit of activity was defined as the amount of enzyme catalyzing the formation of 1 μmol of GS-DNB adduct per minute per milligram of protein, using the molar extinction coefficient of the product (9.6 × 10^3^ L/mol/cm) for calculation.

Carboxylesterase (CarE) activity was measured using a commercial assay kit (Beijing Solarbio Biotechnology Co., Ltd.; Cat# BC0845) with 1-naphthyl acetate as the substrate. Midgut tissues were homogenized in ice-cold extraction buffer using the same Low-temperature grinder as described above. The homogenate was centrifuged at 15,000 rpm for 10 min at 4 °C, and the supernatant was used for the assay. The reaction mixture contained supernatant, reagent I, and reagent II, and absorbance at 450 nm was measured with a SpectraMax M5 microplate reader at 10 s and after 5 min at 37 °C. CarE activity was expressed as U/mg protein, where one unit was defined as the amount of enzyme producing 1 μmol of 1-naphthol per minute per milligram of protein. Hydrolysis of 1-naphthyl acetate releases 1-naphthol, which reacts with fast blue salt to form a colored azo compound for quantification.

The protein concentration of the enzyme supernatant was determined using the BCA Protein Assay Kit (Beijing Solarbio Biotechnology Co., Ltd., Beijing, China, Cat# PC0020). Bovine serum albumin (BSA) provided in the kit (5 mg/mL stock solution) was used as the calibration standard. A standard curve was constructed with BSA concentrations ranging from 0 to 0.5 mg/mL (0, 0.05, 0.1, 0.15, 0.2, 0.3, 0.4, 0.5 mg/mL) prepared by serial dilution with 0.01 M PBS (pH 7.2). The absorbance was measured at 562 nm using a SpectraMax M5 microplate reader after incubation at 37 °C for 30 min.

To validate the role of CarE in β-CYP and *M. anisopliae* tolerance, we conducted synergism bioassays using enzyme-specific inhibitors. A total of 420 healthy adult male German cockroaches were divided into seven groups (60 individuals per group, three biological replicates per group). For carboxylesterase inhibition, 10 μg triphenyl phosphate (TPP, a specific CarE inhibitor) was topically applied to the abdominal tergite 2 h before insecticide or fungal treatment. Mortality was recorded at 16, 24, and 48 h post-treatment, following the same experimental conditions as the main toxicity assay.

### 2.10. Immune Gene Expression Analysis

Samples were collected on days 1–5 post-treatment. Each treatment group consists of 9 insects at each time point, with 3 insects per biological replicate and a total of 3 biological replicates. The untreated control group was sampled immediately before treatment and served as the baseline reference for gene expression analysis. Total RNA was extracted using an RNA Extraction Kit (Sparkjade Biotechnology Co., Ltd., Jinan, China). cDNA was synthesized using Evo M-MLV RT Mix Kit (Accurate Biotechnology). Quantitative real-time PCR was performed using SYBR Green Premix Pro Taq HS qPCR Kit on a Roche LC96 system. Specific primers for *CYP4G19* and *BgPo* genes were designed using Primer Premier 5.0 software, with β-actin as the reference gene. RT-qPCR was performed with three biological replicates derived from the same rearing cohort of cockroaches. Three technical replicates were conducted for each biological sample. Relative expression levels were calculated using the 2^−ΔΔCt^ method.

### 2.11. Statistical Analysis

All data were analyzed using SPSS software (Version 20.0 or 22.0). Compatibility index data were subjected to one-way analysis of variance (ANOVA) followed by Duncan’s multiple range test. Differences in mortality rates among treatment groups were evaluated via one-way ANOVA. LT_50_ values derived from time-mortality bioassays were calculated using Probit regression analysis. For enzyme activity and relative gene expression datasets, one-way ANOVA coupled with the LSD post hoc test was applied for multiple-group comparisons, while independent samples *t*-test was adopted for pairwise comparisons between two groups. All quantitative results were presented as mean ± standard error (SE). A probability value of *p* < 0.05 was defined as the threshold for statistically significant differences.

## 3. Results

### 3.1. Synergistic Effects of β-CYP and M. anisopliae

Acetone showed concentration-dependent inhibitory effects on *M. anisopliae*. Low concentrations (0.5% and 1%) weakly inhibited spore germination and colony growth, while high concentrations (5% and 10%) significantly inhibited mycelial vegetative growth and sporulation (Supplementary Table S1). Compatibility index analysis showed that 0.5% and 1% acetone were compatible with *M. anisopliae* (BI > 66), 5% acetone was moderately toxic (BI = 52), and 10% acetone was toxic (BI = 32) (Supplementary Table S2). Therefore, 1% acetone was selected as the solvent for subsequent experiments.

β-CYP at 1, 5, and 10 μg/mL showed good compatibility with *M. anisopliae* at all tested concentrations (1 × 10^7^, 1 × 10^8^, and 1 × 10^9^ cfu/mL), with all BI values exceeding 66 (Table 1). The lower the β-CYP concentration, the better the compatibility (higher BI values).

### 3.2. Synergistic Effects of β-CYP and M. anisopliae Combinations

Bioassay results showed that both β-CYP and *M. anisopliae* alone were effective against German cockroaches, with mortality increasing in a dose-dependent manner. *M. anisopliae* alone at 1 × 10^7^, 1 × 10^8^, and 1 × 10^9^ cfu/mL resulted in 50%, 72%, and 82% mortality, respectively, with LT_50_ values decreasing from 13.4 to 8.2 days as concentration increased. β-CYP alone at 1, 3, 5, and 7 μg/mL resulted in 15%, 22%, 44%, and 68% mortality, respectively (*p* < 0.05) (Table 2).

Among the combinations, several exhibited synergistic effects. Notably, 1 × 10^7^ cfu/mL *M. anisopliae* with 1 μg/mL β-CYP showed synergism (co-toxicity factor = 35.22); 1 × 10^8^ cfu/mL *M. anisopliae* with 1 μg/mL and 3 μg/mL β-CYP also showed synergistic effects (co-toxicity factors = 25.13 and 22.09, respectively). All combinations resulted in higher mortality than either agent alone, with 1 × 10^8^ and 1 × 10^9^ cfu/mL *M. anisopliae* combined with 7 μg/mL β-CYP achieving 100% mortality.

Importantly, combinations significantly reduced the LT_50_ of *M. anisopliae*. For example, 1 × 10^7^ cfu/mL *M. anisopliae* combined with 1 μg/mL β-CYP reduced LT_50_ from 13.4 to 9.2 days. The shortest LT_50_ (1.7 days) was observed with 1 × 10^9^ cfu/mL *M. anisopliae* combined with 7 μg/mL β-CYP (Table 2).

### 3.3. Histopathological Changes

Based on the significant synergistic effect observed with 1 μg/mL β-CYP and 1 × 10^7^ cfu/mL *M. anisopliae*(co-toxicity factor = 35.22), this combination was selected for histopathological examination at 6 days post-treatment.

**Figure 1:**
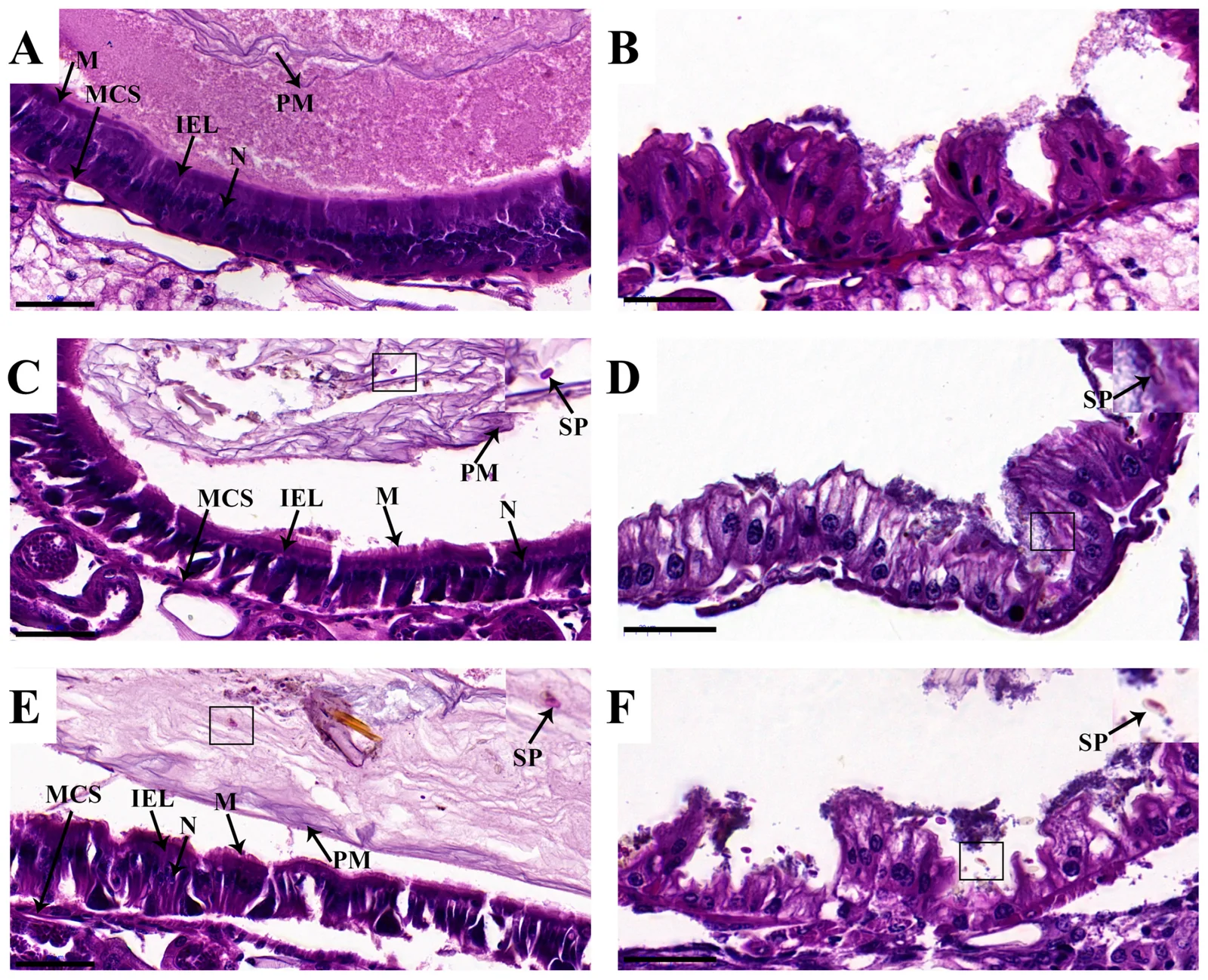
Histopathological sections of midgut and hindgut after 6 days of treatment. (**A**,**B**) β-CYP-treated group; (**C**,**D**) *M. anisopliae*-treated group; (**E**,**F**) combination-treated group. Boxed regions in panels (**C**–**F**) indicate the location of fungal spores. PM: peritrophic membrane; N: nucleus; IEL: intestinal epithelial layer; M: microvilli; MCS: muscle connection system; SP: spores. Scale bar = 50 μm.

**Figure 2:**
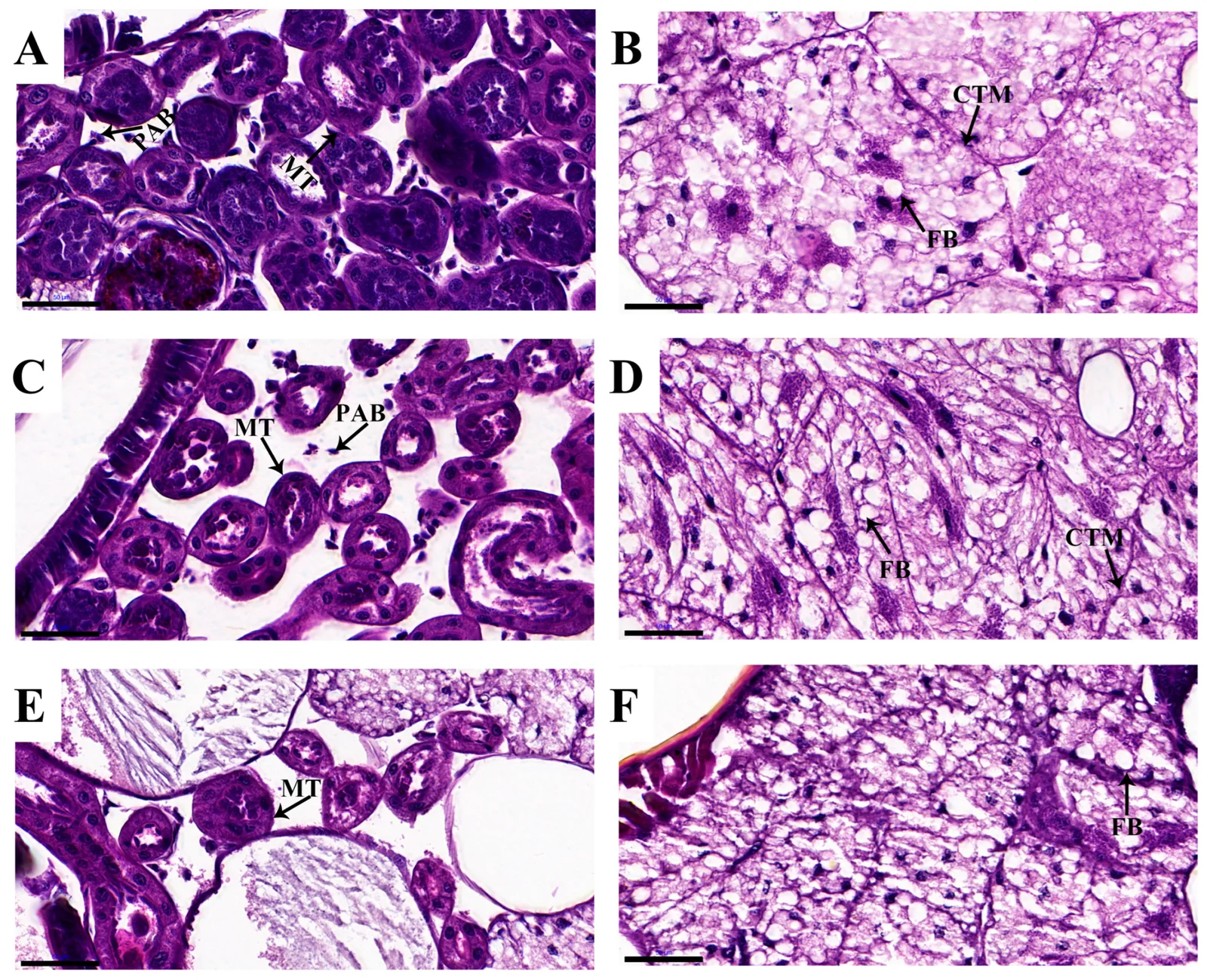
Histopathological sections of Malpighian tubules and fat body after 6 days of treatment. (**A**,**B**) β-CYP-treated group; (**C**,**D**) *M. anisopliae*-treated group; (**E**,**F**) combination-treated group. PAB: Putative apoptotic bodies; MT: Malpighian tubule; CTM: connective tissue membrane; FB: fat body. Scale bar = 50 μm.

In the β-CYP-treated group, slight damage to the peritrophic membrane was observed, whereas no obvious alterations were noted in intestinal epithelium, fat body, or Malpighian tubules (Figure 1A,B and Figure 2A,B). In the *M. anisopliae* alone group, fungal spores were detected in the intestinal wall, with loosening of fat body and Malpighian tubule structure, dilation of Malpighian tubule lumens, increased apoptotic bodies, and blurred boundaries of fat body connective tissue membranes (Figure 1C,D and Figure 2C,D).

In contrast, the combination group exhibited severe tissue damage. The intestines showed disrupted structure with disorganized nuclei, increased vacuoles between epithelial cells, and decreased integrity of the basement membrane–muscle connection system. Malpighian tubule epithelial cells showed contraction and collapse, with reduced diameter. Fat body connective tissue membranes were lysed, and cellular structure became loose. Large numbers of *M. anisopliae* spores were observed throughout the intestines and peritrophic membrane (Figure 1E,F and Figure 2E,F).

### 3.4. Relative Abundance of M. anisopliae

**Figure 3:**
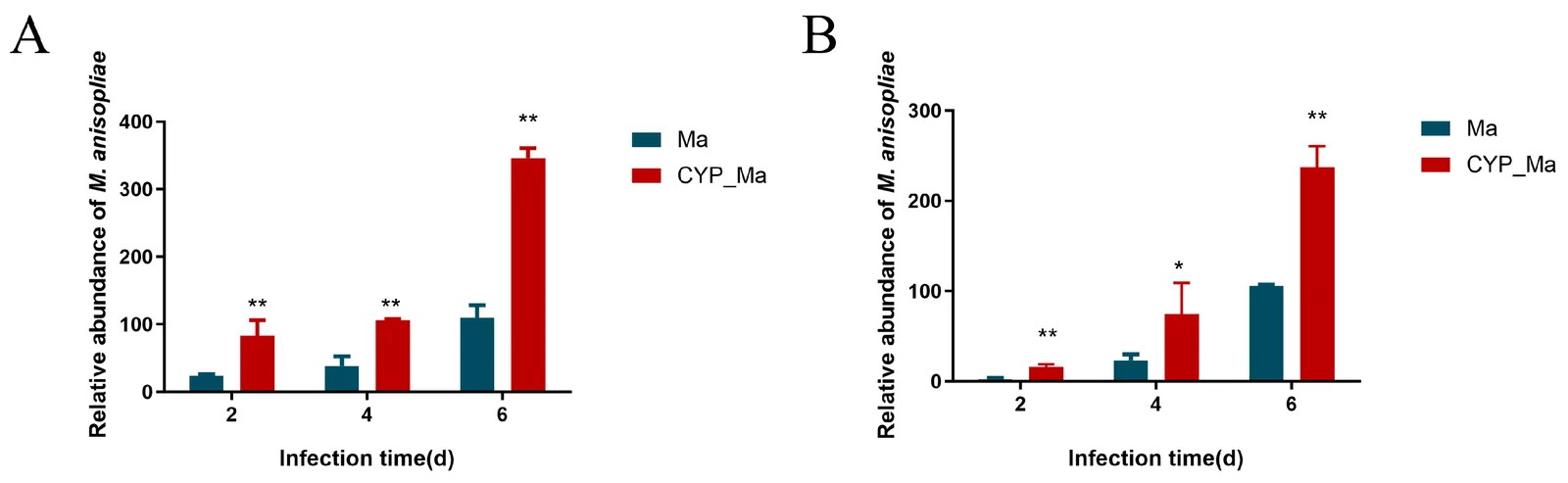
Relative abundance of *M. anisopliae* in (**A**) gut and (**B**) hemolymph. Ma: *M. anisopliae* alone group; CYP_Ma: combination group. * *p* < 0.05; ** *p* < 0.01.

Quantitative real-time PCR analysis revealed that the combination treatment significantly promoted *M. anisopliae* proliferation compared to fungal treatment alone. This effect became increasingly pronounced over time. In the combination group at day 6, the relative abundance of *M. anisopliae* in the gut and hemolymph reached 346.28-fold and 237.44-fold of the control group, respectively, with highly significant differences (*P* < 0.01) (Figure 3).

### 3.5. Gut Microbiota Alterations Induced by β-CYP and M. anisopliae Treatment

**Figure 4:**
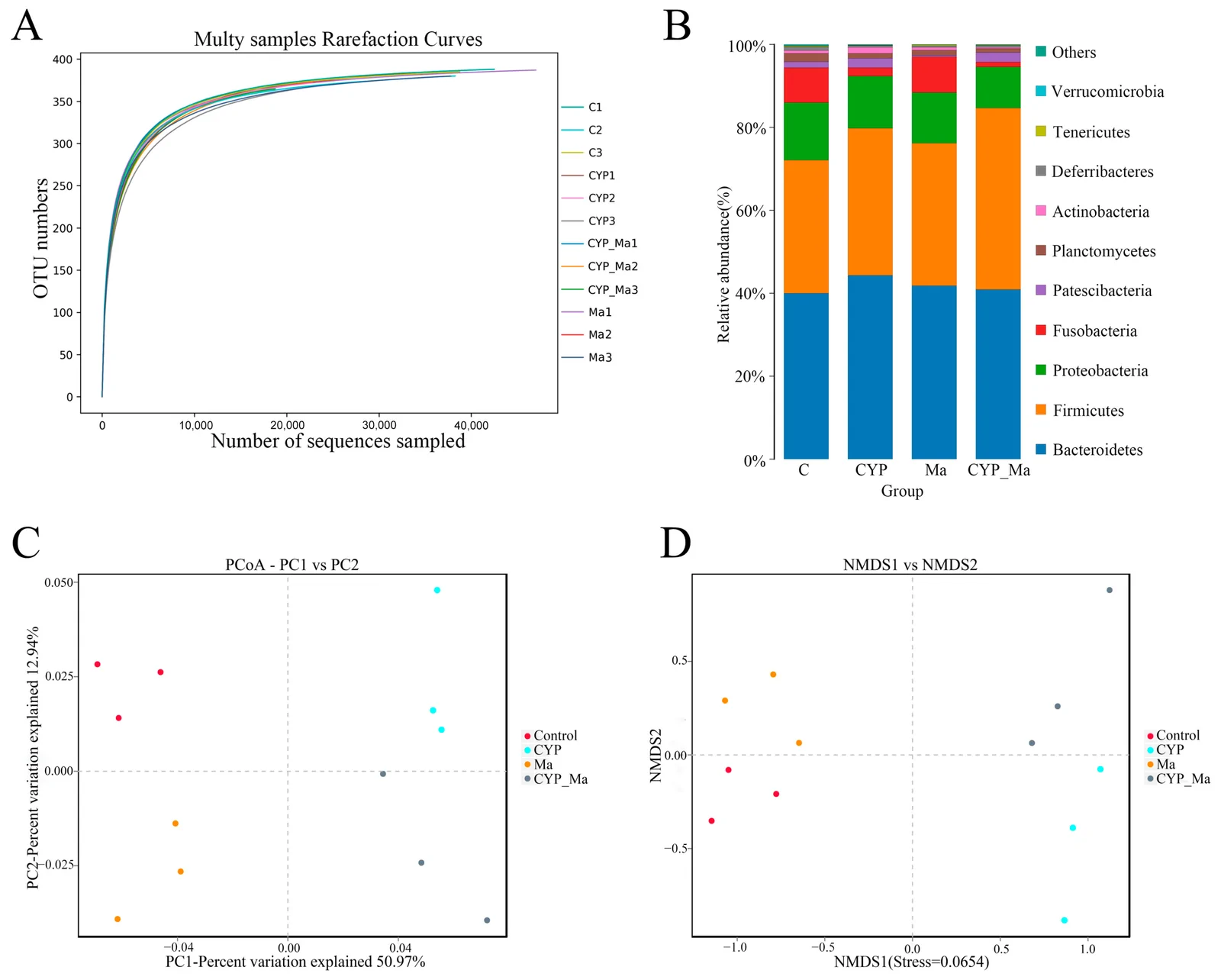
Gut microbiota analysis. (**A**) Dilution curves; (**B**) Phylum-level composition; (**C**) PCoA plot; (**D**) NMDS plot. C: control; Ma: *M. anisopliae* alone; CYP: β-CYP alone; CYP_Ma: combination.

High-throughput sequencing of 16S rRNA yielded high-quality sequences with Good’s coverage averaging 99%, indicating reliable sequencing results (Figure 4A). Alpha diversity indices showed that the combination treatment reduced OTU numbers and Shannon diversity index compared to the control group, although some differences did not reach statistical significance (Supplementary Table S3). PCoA and NMDS analyses revealed clear separation among the four treatment groups, with the combination group samples farthest from the control group, indicating the most substantial alteration of gut microbial community structure (Figure 4C,D).

At the phylum level, Bacteroidetes dominated in the control, *M. anisopliae* alone, and β-CYP alone groups, while this dominance was replaced by Firmicutes in the combination group (Figure 4B). This remodeling of the core flora structure was significant (*p* < 0.05). At the genus level, the combination treatment induced marked changes in microbial composition (Table 3). Compared to the control group, the combination group showed significant increases in the relative abundance of opportunistic pathogens *Weissella* (*p* < 0.05) and *Alistipes* (*p* < 0.05), while the relative abundances of beneficial bacteria Christensenellaceae_R-7_group (*p* < 0.05), *Enterococcus* (*p* < 0.01), and *Fusobacterium* (*p* < 0.05) decreased significantly. *Weissella* emerged as the dominant genus in the combination group.

### 3.6. Contribution of Weissella to Combination Efficacy

**Figure 5:**
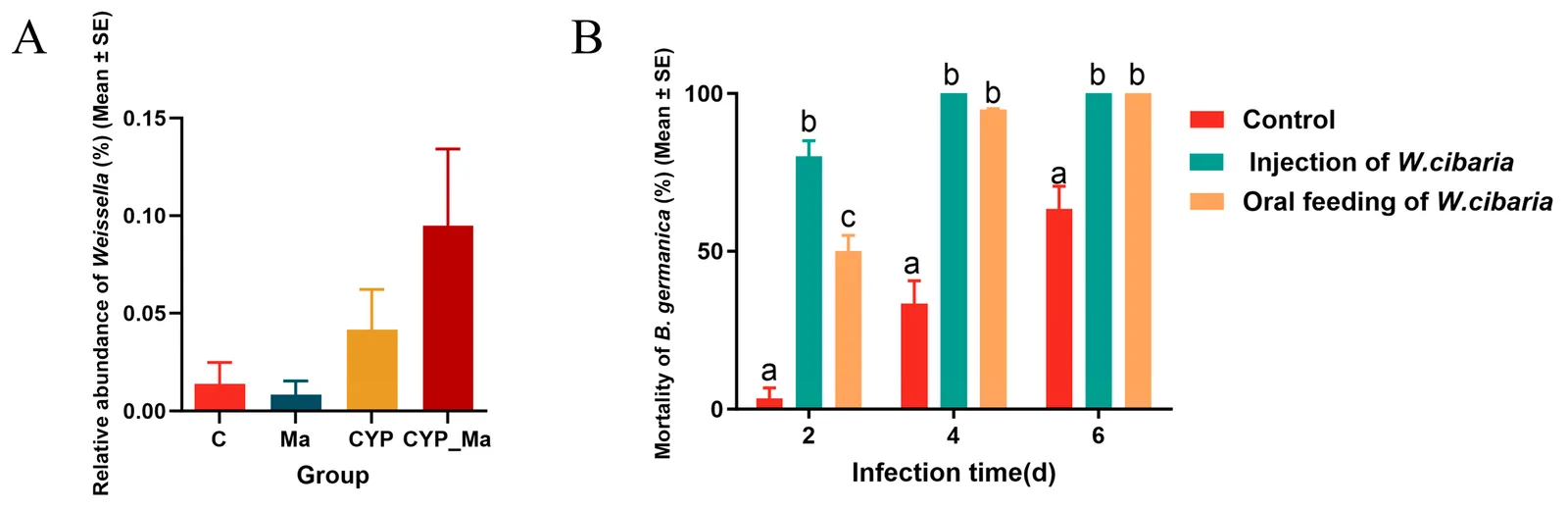
Effect of *Weissella* on combination treatment efficacy. (**A**) Relative abundance of *Weissella* at genus level. (**B**) Mortality of *B. germanica* after *Weissella* recolonization followed by combination treatment. Different lowercase letters indicate significant differences among treatment groups at the same time point (*p* < 0.05),where applicable.

Given the specific increase in *Weissella* abundance in the combination group (Figure 5A), recolonization experiments were performed. After antibiotic depletion of gut microbiota, *Weissella* was reintroduced via oral feeding or hemocoel injection, followed by combination treatment.

At day 2 post-treatment, mortality reached 50% in the orally fed group and 80% in the hemocoel injection group, significantly higher than the control group (*p* < 0.05). By day 4, both recolonization groups exceeded 90% mortality, and by day 6, both reached 100% mortality, while the control group (no *Weissella* recolonization) showed only 63.33% mortality (*p* < 0.05). Notably, hemocoel injection resulted in faster death compared to oral administration (Figure 5B).

### 3.7. Detoxification Enzyme Activities

**Figure 6:**
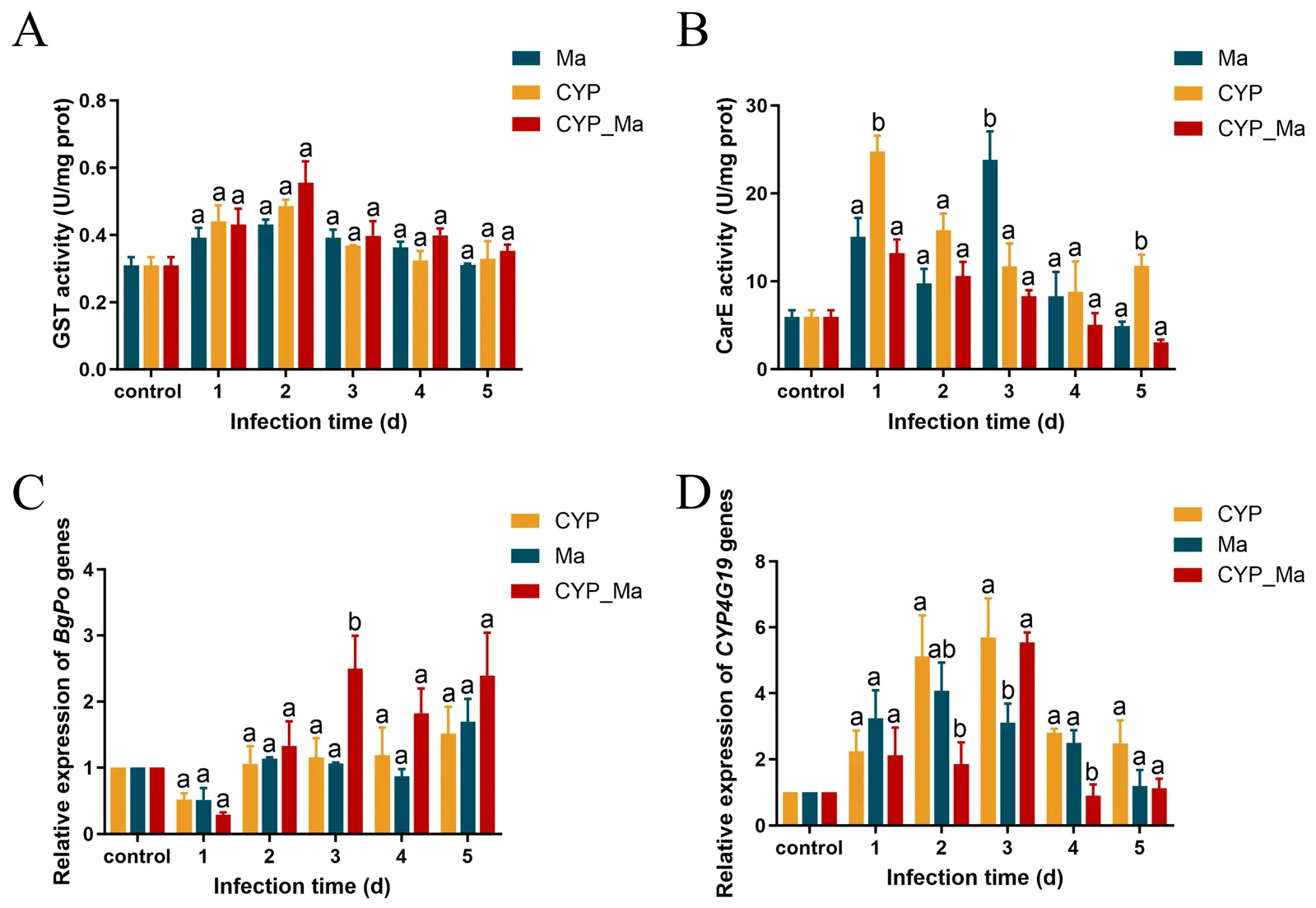
Effects of different treatments on detoxification enzyme activities and immune gene expression. (**A**) Glutathione S-transferase; (**B**) Carboxylesterase; (**C**) *BgPo* expression; (**D**) *CYP4G19* expression. Different lowercase letters indicate significant differences among treatment groups at the same time point (*p* < 0.05),where applicable.

Both β-CYP and *M. anisopliae* alone induced increases in glutathione S-transferase and carboxylesterase activities, whereas the combination treatment significantly altered these detoxification responses (Figure 6).

Glutathione S-transferase activity showed an initial increase followed by a decrease in all groups, with no significant differences among groups (Figure 6A). Carboxylesterase activity was activated earlier than other enzymes. In the combination group, carboxylesterase activity gradually decreased from days 1 to 5 and was lower than that in the β-CYP-alone group at days 1, 3, and 5 (Figure 6B).

**Figure 7:**
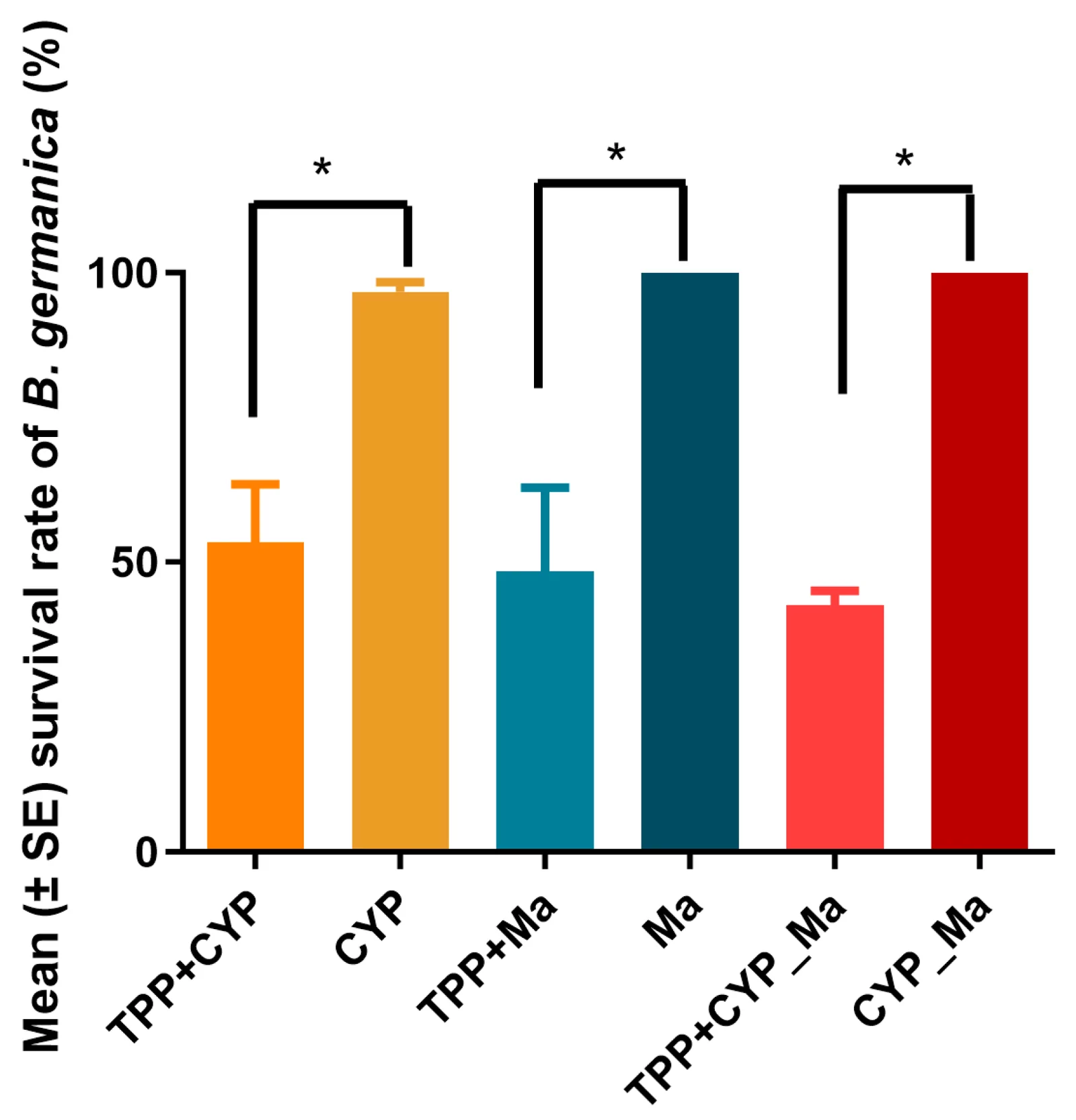
Effect of triphenyl phosphate inhibition on the survival of *B. germanica* under different treatments. Asterisks (*) indicate significant differences between the indicated groups (*p* < 0.05).

The triphenyl phosphate inhibition assay further supported the involvement of carboxylesterase in the response to β-CYP and *M. anisopliae*. Addition of the carboxylesterase inhibitor significantly reduced survival rates in all treatment groups, with the combination group showing the lowest survival (42.5%) (*p* < 0.05) (Figure 7).

Field resistance surveillance on wild *B. germanica* populations from urban hospital habitats in Iran has proven that overexpressed carboxylesterase and cytochrome P450 enzymes constitute the dominant metabolic resistance mechanism against commonly used synthetic insecticides. Consistently, our results verified that the synergistic effect of *M. anisopliae* and β-CYP could effectively restrain the upregulation of key detoxification enzymes. These findings provide a basis for further evaluating the potential of the β-CYP–*M. anisopliae* combination against pyrethroid-resistant field cockroach populations.

### 3.8. Immune-Related Gene Expression

Gene expression levels were normalized to β-actin and expressed as fold changes relative to the corresponding control group using the 2^−ΔΔCt^ method. Expression of the phenoloxidase gene *BgPo* was significantly upregulated in the combination group at days 3 and 5 post-treatment (2.49-fold and 2.39-fold of the control, respectively). In contrast, the *M. anisopliae* alone group showed significant upregulation only at day 5 (1.7-fold), while the β-CYP alone group showed no significant changes (Figure 6C). These results indicate that the combination treatment advanced the activation of the phenoloxidase cascade.

The *CYP4G19* gene was significantly upregulated in both single-treatment groups. In β-CYP alone and *M. anisopliae* alone groups, *CYP4G19* expression peaked at days 2–3 (5.11-fold and 5.69-fold of control, respectively). However, in the combination group, *CYP4G19* was significantly upregulated only at day 3 (5.54-fold), returning to control levels by days 4–5. The combination group showed significantly lower *CYP4G19* expression than the β-CYP alone group at day 2 (*p* < 0.05) and significantly lower than both single-treatment groups at day 4 (*p* < 0.05) (Figure 6D).

## 4. Discussion

This study demonstrates that specific combinations of β-CYP and *M. anisopliae* produces synergistic effects against *B. germanica*, substantially increasing mortality and reducing LT_50_ compared to either treatment alone. This finding is consistent with previous reports of insecticide-fungus synergy in various pests. The present study extends these observations to a major urban pest and provides comprehensive mechanistic insights from multiple dimensions.

The synergy involves multiple complementary mechanisms. First, the combination accelerates fungal infection and tissue destruction. Histopathology showed slight peritrophic membrane damage in the β-CYP-treated group, whereas the combination treatment was associated with more extensive tissue damage and *M. anisopliae* alone showed limited tissue invasion by day 6, the combination group exhibited extensive destruction of midgut, hindgut, Malpighian tubules, and fat body, with abundant fungal spores visible in tissues. This accelerated pathology correlated with significantly higher fungal abundance in both hemolymph and gut tissues of combination-treated cockroaches. It is therefore hypothesized that β-CYP promotes fungal entry and proliferation by compromising cuticular integrity. This is similar to results showing that combining *M. anisopliae* with abamectin increased beetle mortality, and combining *M. anisopliae* with chlorantraniliprole increased locust mortality. This suggests that the dysregulation of the immune system and detoxification system under combination treatment, along with the development of opportunistic bacteria, may be among the causes of the synergistic effect [39].

Second, the combination treatment induced severe gut microbiota dysbiosis, which may represent another key driver of the synergistic effect. The present study found that the combination treatment led to Firmicutes replacing Bacteroidetes as the dominant phylum, accompanied by significant enrichment of opportunistic pathogens (*Weissella*, *Alistipes*), while beneficial bacteria with antifungal activity or roles in maintaining gut homeostasis (e.g., *Enterococcus*, *Fusobacterium*) were greatly reduced. *Weissella* emerged as the dominant genus in the combination group, and its function was validated through recolonization experiments: reintroduction of *Weissella* significantly increased the lethality of the combination treatment against cockroaches, with hemocoel injection being more effective than oral administration. This result strongly suggests that the gut barrier disruption caused by both the chemical insecticides and fungal infection may enable opportunistic pathogens (such as *Weissella*) resident in the gut to translocate to the hemocoel, thereby exacerbating systemic infection and toxemia. Previous studies have shown that the gut microbiota of *B. germanica* plays an important role in defending against entomopathogenic fungi and chemical toxins [40,41,42]; our results confirm, from the opposite perspective, the detrimental impact of gut dysbiosis on host health.

Furthermore, the combination treatment suppressed host detoxification and immune defense capabilities. We found that although β-CYP or *M. anisopliae* alone induced increases in GST and CarE activities, the combination treatment significantly suppressed this induction. After specific inhibition of CarE activity using triphenyl phosphate, survival rates decreased significantly in all treatment groups, with the combination group showing the greatest reduction, confirming the central role of CarE in the detoxification process. This suggests that the combination treatment may interfere, through an unknown mechanism, with the detoxification enzyme induction pathways that respond to chemical insecticides and fungal toxins, thereby compromising host chemical defense. At the immune level, the combination treatment advanced and enhanced the expression of the phenoloxidase gene *BgPo*, reflecting host response to earlier and more aggressive fungal infection; however, expression of the *CYP4G19* gene, which is involved in cuticular hydrocarbon synthesis, was significantly suppressed in the later stage of combination treatment. Downregulation of *CYP4G19* may affect cuticular hydrocarbon metabolism and potentially compromise cuticular barrier function, which could contribute to increased susceptibility to subsequent fungal invasion or insecticide exposure. Because the control group was not sampled independently at each day in the gene-expression experiment, the observed temporal patterns should be interpreted cautiously.

Several limitations of the present study should be acknowledged. First, the experiments were conducted using a susceptible laboratory strain of *B. germanica*, and the efficacy and underlying mechanisms of the β-CYP–*M. anisopliae* combination may differ among field populations with different resistance phenotypes. Field investigations on wild cockroach populations have linked metabolic enzyme-mediated resistance phenotypes to insecticide performance [43], further demonstrating the prospect of our combined fungus-insecticide strategy for overcoming metabolic resistance. Second, an untreated or vehicle-treated control was not included in the histopathological experiment; therefore, the observed tissue alterations, particularly the slight peritrophic membrane damage in the β-CYP-treated group, should be interpreted cautiously. Third, for gene-expression analysis, the untreated control was sampled before treatment (day 0) and used as the baseline reference, whereas treatment groups were sampled on days 1–5; thus, the contribution of natural temporal variation in gene expression cannot be completely excluded. Finally, although the enrichment of *Weissella* was associated with enhanced mortality following combination treatment, its precise causal role in gut barrier disruption and systemic infection remains to be established. Further studies using field-derived resistant populations, time-matched controls, and direct measurements of microbial translocation will help validate these findings under field-relevant conditions.

## 5. Conclusions

In conclusion, the combination of β-CYP and *M. anisopliae* achieves high synergistic efficacy against *B. germanica* by launching a multi-faceted, coordinated attack—including accelerated fungal proliferation, induction of gut dysbiosis and opportunistic pathogen translocation, suppression of detoxification enzyme activities, and compromise of immune defense. This multi-target mechanism makes it difficult for pests to evade through a single resistance mechanism, rendering resistance evolution significantly more challenging than with single agents. This strategy effectively combines the high efficacy and rapid action of chemical insecticides with the environmental friendliness and low resistance potential of entomopathogenic fungi, achieving complementary advantages.

This study provides a solid theoretical foundation for developing integrated pest management strategies that reduce chemical insecticide usage while enhancing biocontrol efficacy. Future research should focus on field validation, formulation optimization, and evaluation of the long-term effects of this strategy on resistance evolution.

## Figures and Tables

**Table 1:** Compatibility between β-CYP and *M. anisopliae*.

Ma (cfu/mL)	Treatment		Conidia Germination Inhibition Rate (%)	Colony Diameter Inhibition Rate (%)			Number of Produced Conidia Inhibition Rate (%)
	Acetone	β-CYP (μg/mL)		5 d	10 d	15 d	
1 × 10^7^	1%	0	1.56 ± 3.21 a	0.37 ± 0.63 a	5.64 ± 0.59 a	3.24 ± 2.20 a	16.63 ± 3.62 a
	0	1	1.44 ± 2.78 a	0.73 ± 4.44 a	3.08 ± 0.89 a	3.24 ± 1.59 a	21.18 ± 2.13 a
	0	5	1.92 ± 2.81 a	5.11 ± 0.73 a	8.72 ± 1.78 a	7.43 ± 2.86 a	25.18 ± 1.07 a
	0	10	8.26 ± 5.63 a	5.84 ± 1.26 a	7.69 ± 2.96 a	7.43 ± 7.74 a	38.28 ± 8.02 a
1 × 10^8^	1%	0	1.86 ± 1.27 a	2.13 ± 2.13 a	2.65 ± 1.83 a	2.77 ± 2.71 a	16.11 ± 1.31 a
	0	1	1.86 ± 1.75 a	4.26 ± 1.23 a	4.76 ± 1.62 a	2.67 ± 0.49 a	38.33 ± 0.96 ab
	0	5	2.57 ± 1.23 a	5.32 ± 1.84 a	5.47 ± 0.93 a	4.69 ± 10.42 a	45.00 ± 0.00 ab
	0	10	7.11 ± 0.65 a	5.32 ± 0.61 a	8.99 ± 1.22 a	6.50 ± 0.21 a	50.33 ± 1.44 b
1 × 10^9^	1%	0	2.51 ± 1.31 a	2.38 ± 1.72 a	3.05 ± 2.03 a	2.19 ± 0.00 a	28.68 ± 8.95 a
	0	1	1.66 ± 1.46 a	1.66 ± 1.04 a	3.52 ± 1.01 a	4.38 ± 0.84 a	40.56 ± 6.95 ab
	0	5	3.55 ± 0.14 a	5.00 ± 0.71 a	10.55 ± 2.90 a	9.00 ± 0.64 a	53.32 ± 5.01 b
	0	10	6.03 ± 5.33 a	5.00 ± 0.71 a	11.22 ± 3.55 a	9.73 ± 3.82 a	53.49 ± 4.98 b

Note: Data are presented as mean ± SE. Different letters within the same *M. anisopliae* concentration group indicate significant differences (*p* < 0.05, Duncan’s test).

**Table 2:** Virulence of β-CYP, *M. anisopliae*, and their combinations against *B. germanica*.

Treatment		*n*	% Mortality ± SE	LT_50_ (95% CI) d	Slope ± SE	Χ^2^	Co-Toxicity Factor	Effect
Ma (cfu/mL)	β-CYP (μg/mL)							
1 × 10^7^	0	60	50 ± 5 abc	13.4 (12.4–14.6)	0.16 ± 0.02	12.04		
1 × 10^8^	0	60	72 ± 3 bc	10.0 (9.1–11.0)	0.21 ± 0.01	40.51		
1 × 10^9^	0	60	82 ± 4 cd	8.2 (7.1–9.3)	0.21 ± 0.01	60.93		
0	1	60	15 ± 3 a	NA	NA	NA		
0	3	60	22 ± 7 a	NA	NA	NA		
0	5	60	44 ± 10 ab	NA	NA	NA		
0	7	60	68 ± 3 bc	2.4 (0.9–3.8)	0.05 ± 0.10	49.56		
1 × 10^7^	1	60	78 ± 7 bc	9.2 (8.4–9.9)	0.20 ± 0.02	18.52	35.22	synergistic
	3	60	70 ± 5 a	6.9 (5.5–8.2)	0.12 ± 0.02	8.56	15.07	additive
	5	60	83 ± 6 abc	6.4 (5.7–7.0)	0.14 ± 0.01	10.77	15.74	additive
	7	60	92 ± 3 bc	5.0 (3.7–6.0)	0.14 ± 0.01	13.00	8.92	additive
1 × 10^8^	1	60	95 ± 5 bc	5.2 (3.9–6.3)	0.26 ± 0.12	98.30	25.13	synergistic
	3	60	95 ± 5 bc	5.5 (4.8–6.0)	0.22 ± 0.02	16.26	22.09	synergistic
	5	60	97 ± 3 bc	3.6 (2.8–4.2)	0.45 ± 0.04	4.96	14.90	additive
	7	60	100 ± 0 c	3.0 (2.4–3.5)	0.33 ± 0.03	6.60	9.86	additive
1 × 10^9^	1	60	97 ± 2 bc	5.6 (4.4–6.9)	0.30 ± 0.01	117.72	14.52	additive
	3	60	97 ± 2 bc	5.2 (4.4–6.0)	0.27 ± 0.01	39.02	12.88	additive
	5	60	95 ± 0 bc	5.0 (3.5–6.3)	0.28 ± 0.02	104.65	5.87	additive
	7	60	100 ± 0 c	1.7 (1.2–2.2)	0.39 ± 0.04	2.94	6.16	additive

Note: The fungal concentrations are reported as the concentrations of the prepared suspensions (cfu/mL) to maintain consistency with the compatibility assays and other experiments. A topical application volume of 2 μL per insect corresponds to 2 × 10^4^, 2 × 10^5^, and 2 × 10^6^ cfu/insect for suspensions of 1 × 10^7^, 1 × 10^8^, and 1 × 10^9^ cfu/mL, respectively. The solvent control consisted of 2 μL of 1% acetone. Different letters indicate significant differences among groups (*p* < 0.05, Duncan’s test). *n* = 60 (20 per group, three independent replicates). NA: not applicable.

**Table 3:** Differential analysis of relative abundance of intestinal bacteria at genus level.

Genus	CYP_Ma			CYP		Ma
	C	Ma	CYP	C	Ma	C
Alistipes	0.065 *	0.052	0.020	0.085 **	0.072 *	0.013
Christensenellaceae_R-7_group	0.025 *	0.015	0.020 *	0.005	0.005	0.010
Enterococcus	0.020 **	0.010	0.017 **	0.003	0.007	0.010
Fusobacterium	0.063 *	0.071 *	0.007	0.055	0.063 *	0.008
Lachnoclostridium	0.037 *	0.029 *	0.018	0.018	0.011	0.008
Paludibacter	0.010	0.029 ***	0.004	0.006	0.025 ***	0.018 **
Tyzzerella_3	0.017	0.012	0.025	0.042 *	0.012	0.030
Weissella	0.081 *	0.087 **	0.053	0.028	0.033	0.006

Note: Values represent the difference in relative abundance between the first row group and the second row group. “Red” indicates increase in CYP_Ma; “Blue” indicates decrease in CYP_Ma. * *p* < 0.05, ** *p* < 0.01, *** *p* < 0.001 (ANOVA). C: control; Ma: *M. anisopliae* alone; CYP: β-CYP alone; CYP_Ma: combination.

## Data Availability

Raw gut microbiota sequencing data generated in this study have been deposited in the NCBI Sequence Read Archive (SRA) under accession number BioProject PRJNA1452616. All other experimental data are provided within the article and Supplementary Materials.

## Supplementary Materials

The following supporting information can be downloaded at https://www.mdpi.com/article/10.3390/insects17090896/s1: Supplementary Table S1. Effects of acetone on spore germination, colony diameter, and sporulation of *M. anisopliae*. Supplementary Table S2. Compatibility index analysis of acetone and beta-cypermethrin with *M. anisopliae*. Supplementary Table S3. Operational taxonomic unit (OTU) numbers and alpha diversity indices of gut microbiota for each sample.

## Abbreviations

The following abbreviations are used in this manuscript:

β-CYP	Beta-cypermethrin
Met-Hybrid	Transgenic *Metarhizium pinghaense* expressing spider neurotoxin
SD strain	Susceptible laboratory strain
LT_50_	Median lethal time
qRT-PCR	Quantitative real-time PCR
OTUs	Operational taxonomic units
SRA	Short Read Archive
GST	Glutathione S-transferase
CDNB	1-chloro-2,4-dinitrobenzene
CarE	Carboxylesterase
BSA	Bovine serum albumin
PM	Peritrophic membrane
N	Nucleus
IEL	Intestinal epithelial layer
M	Microvilli
MCS	Muscle connection system
S	Spore
PABs	Putative apoptotic bodies
CTM	Connective tissue membrane
FB	Fat body
CYP_Ma	Combination group
C	Control
CYP	β-CYP alone

## References

[1] Motevalli-Haghi S.F., Shemshadian A., Nakhaei M., Faridnia R., Dehghan O., Malekzadeh Shafaroudi M., Nejadi Kelarijani M., Nikookar S.H., Kalani H., Fakhar M. (2021). First report of *Lophomonas* spp. in German cockroaches (*Blattella germanica*) trapped in hospitals, northern Iran. J. Parasit. Dis..

[2] Salehzadeh A., Tavacol P., Mahjub H. (2007). Bacterial, fungal and parasitic contamination of cockroaches in public hospitals of Hamadan, Iran. J. Vector Borne Dis..

[3] Wu X., Appel A.G. (2017). Insecticide resistance of several field-collected German cockroach (Dictyoptera: Blattellidae) strains. J. Econ. Entomol..

[4] Fardisi M., Gondhalekar A.D., Scharf M.E. (2017). Development of diagnostic insecticide concentrations and assessment of insecticide susceptibility in German cockroach (Dictyoptera: Blattellidae) field strains collected from public housing. J. Econ. Entomol..

[5] Pasay C., Arlian L., Morgan M., Gunning R., Rossiter L., Holt D., Walton S., Beckham S., McCarthy J. (2009). The effect of insecticide synergists on the response of scabies mites to pyrethroid acaricides. PLoS Negl. Trop. Dis..

[6] Chang K.S., Shin E.H., Jung J.S., Park C., Ahn Y.-J. (2010). Monitoring for insecticide resistance in field-collected populations of *Blattella germanica* (Blattaria: Blattellidae). J. Asia-Pac. Entomol..

[7] Resti R., Intan A., Muhammad Zai Halifiah S., Risa Ukhti M., Robby J. (2025). Status and Mechanism of Insecticide Resistance in German Cockroach (*Blattella germanica* L.) Worldwide: A Literature Review. Trop. Life Sci. Res..

[8] Siegfried B.D., Scott J.G. (1992). Insecticide Resistance Mechanisms in the German Cockroach, Blattella germanica (L.).

[9] Dehkordi A.S., Abadi Y.S., Nasirian H., Hazratian T., Gorouhi M.A., Yousefi S., Paksa A. (2017). Synergists action of piperonyl butoxide and S,S,S-tributyl phosphorotrithioate on toxicity of carbamate insecticides against *Blattella germanica*. Asian Pac. J. Trop. Med..

[10] Wei Y., Appel A.G., Moar W.J., Liu N. (2001). Pyrethroid resistance and cross-resistance in the German cockroach, *Blattella germanica* (L). Pest. Manag. Sci..

[11] Appel A.G. (1992). Performance of gel and paste bait products for German cockroach (Dictyoptera: Blattellidae) control: Laboratory and field studies. J. Econ. Entomol..

[12] Hamilton J.A., Wada-Katsumata A., Schal C. (2023). Cockroaches as trojan horses for control of cockroach aggregations with baits. J. Econ. Entomol..

[13] Appel A.G. (2003). Laboratory and field performance of an indoxacarb bait against German cockroaches (Dictyoptera: Blattellidae). J. Econ. Entomol..

[14] Lee S.-H., Choe D.-H., Rust M.K., Lee C.-Y. (2022). Reduced susceptibility towards commercial bait insecticides in field German cockroach (Blattodea: Ectobiidae) populations from California. J. Econ. Entomol..

[15] Lucero I.M., Gaire S., DeVries Z.C. (2025). Effects of age and environmental conditions on cockroach gel bait performance. J. Econ. Entomol..

[16] Lopes R., Alves S.B. (2011). Differential susceptibility of adults and nymphs of *Blattella germanica* (L.) (Blattodea: Blattellidae) to infection by *Metarhizium anisopliae* and assessment of delivery strategies. Neotrop. Entomol..

[17] Zhang X.C., Li X.X., Gong Y.W., Li Y.R., Zhang K.L., Huang Y.H., Zhang F. (2018). Isolation, identification, and virulence of a new *Metarhizium anisopliae* strain on the German cockroach. J. Econ. Entomol..

[18] de Faria M.R., Wraight S.P. (2007). Mycoinsecticides and mycoacaricides: A comprehensive list with worldwide coverage and international classification of formulation types. Biol. Control.

[19] Li Z., Alves S.B., Roberts D.W., Fan M., Delalibera I., Tang J., Lopes R.B., Faria M., Rangel D.E. (2010). Biological control of insects in Brazil and China: History, current programs and reasons for their successes using entomopathogenic fungi. Biocontrol. Sci. Techn..

[20] Rust M.K. (2021). Alternative control measures. Biology and Management of the German Cockroach.

[21] Schrank A., Vainstein M.H. (2010). *Metarhizium anisopliae* enzymes and toxins. Toxicon.

[22] Ruiz-Sanchez E., Lange A.B., Orchard I. (2010). Effects of the mycotoxin destruxin A on *Locusta migratoria* visceral muscles. Toxicon.

[23] Gutierrez A.C., Gołębiowski M., Pennisi M., Peterson G., García J.J., Manfrino R.G., López Lastra C.C. (2015). Cuticle fatty acid composition and differential susceptibility of three species of cockroaches to the entomopathogenic fungi *Metarhizium anisopliae* (Ascomycota, Hypocreales). J. Econ. Entomol..

[24] Wang C., Wang S. (2017). Insect pathogenic fungi: Genomics, molecular interactions, and genetic improvements. Annu. Rev. Entomol..

[25] Aw K.M.S., Hue S.M. (2017). Mode of infection of *Metarhizium* spp. fungus and their potential as biological control agents. J. Fungi.

[26] Paula A.R., Carolino A.T., Paula C.O., Samuels R.I. (2011). The combination of the entomopathogenic fungus *Metarhizium anisopliae* with the insecticide Imidacloprid increases virulence against the dengue vector *Aedes aegypti* (Diptera: Culicidae). Parasites Vectors.

[27] Qayyum M.A., Saleem M.A., Saeed S., Wakil W., Ishtiaq M., Ashraf W., Ahmed N., Ali M., Ikram R.M., Yasin M. (2020). Integration of entomopathogenic fungi and eco-friendly insecticides for management of red palm weevil, *Rhynchophorus ferrugineus* (Olivier). Saudi J. Biol. Sci..

[28] Liu S., Zhang L., Xu J., Jiang X., Zheng N., Yu Y. (2021). Synergetic Effects of Sublethal Dosage of Beta-cypermethrin on *Metarhizium rileyi* against *Spodoptera litura*. J. Henan Agric. Sci..

[29] Jia M., Cao G., Li Y., Tu X., Wang G., Nong X., Whitman D.W., Zhang Z. (2016). Biochemical basis of synergism between pathogenic fungus *Metarhizium anisopliae* and insecticide chlorantraniliprole in *Locusta migratoria* (Meyen). Sci. Rep..

[30] Zhang F., Sun X.X., Zhang X.C., Zhang S., Lu J., Xia Y.M., Huang Y.H., Wang X.J. (2018). The interactions between gut microbiota and entomopathogenic fungi: A potential approach for biological control of *Blattella germanica* (L.). Pest Manag. Sci..

[31] Chao Y., Wang M., Dai W., Dong F., Wang X., Zhang F. (2020). Synergism between Hydramethylnon and *Metarhizium anisopliae* and Their Influence on the Gut Microbiome of *Blattella germanica* (L.). Insects.

[32] Yang R., Zhang M., Schal C., Jiang M., Cai T., Zhang F. (2021). Boric acid enhances *Metarhizium anisopliae* virulence in *Blattella germanica* (L.) by disrupting the gut and altering its microbial community. Biol. Control.

[33] Zhang X.C., Jiang M., Zang Y.N., Zhao H.Z., Liu C.X., Liu B.R., Xue H., Schal C., Lu X.M., Zhao D.Q. (2022). *Metarhizium anisopliae* is a valuable grist for biocontrol in beta-cypermethrin-resistant *Blattella germanica* (L.). Pest Manag. Sci..

[34] Zhao H., Jiang M., Wang X., Gao H., Zhang Y., Wang J., Zhang X., Zhao D., Zhang F. (2024). Pathogenic fungi synergistically cooperate with *Serratia marcescens* to increase cockroach mortality. Pestic. Biochem. Physiol..

[35] Rossi-Zalaf L., Alves S., Lopes R., Silveira Neto S., Tanzini M. (2008). Interação de microrganismos com outros agentes de controle de pragas e doenças. Controle Microbiano Pragas Am. Lat. Avanços Desafios.

[36] Mansour N., Eldefrawi M., Toppozada A., Zeid M. (1966). Toxicological studies on the Egyptian cotton leaf worm, *Prodenia litura*. VI. Potentiation and antagonism of organophosphorus and carbamate insecticides. J. Econ. Entomol..

[37] Duan Y., Wu H., Ma Z., Yang L., Ma D. (2017). Scanning electron microscopy and histopathological observations of *Beauveria bassiana* infection of Colorado potato beetle larvae. Microb. Pathog..

[38] Livak K.J., Schmittgen T.D. (2001). Analysis of relative gene expression data using real-time quantitative PCR and the 2− ΔΔCT method. Methods.

[39] Kryukov V.Y., Rotskaya U., Yaroslavtseva O., Polenogova O., Kryukova N., Akhanaev Y., Krivopalov A., Alikina T., Vorontsova Y.L., Slepneva I. (2021). Fungus *Metarhizium robertsii* and neurotoxic insecticide affect gut immunity and microbiota in Colorado potato beetles. Sci. Rep..

[40] Jian Z., Guang-ming C., Ying-hua W., Guo-dong L., Han-hong X. (2022). Effect of rotenone on the gut microbiota of *Blattella germanica*. Chin. J. Vector Biol. Control.

[41] Oh S.N., Seo M.J., Youn Y.N., Yu Y.M. (2015). Antifungfal activity against plant pathogenic fungi on insect enterobacteriaceae. Korean J. Pestic. Sci..

[42] Zhang F., Huang Y.H., Liu S.Z., Zhang L., Li B.T., Zhao X.X., Fu Y., Liu J.J., Zhang X.X. (2013). *Pseudomonas reactans*, a bacterial strain isolated from the intestinal flora of *Blattella germanic* a with anti-Beauveria bassiana activity. Environ. Entomol..

[43] Salehi A., Vatandoost H., Hazratian T., Sanei-Dehkordi A., Hooshyar H., Arbabi M., Salim-Abadi Y., Sharafati-Chaleshtori R., Gorouhi M.A., Paksa A. (2016). Detection of Bendiocarb and Carbaryl Resistance Mechanisms among German Cockroach *Blattella germanica* (Blattaria: Blattellidae) collected from Tabriz Hospitals, East Azerbaijan Province, Iran in 2013. J. Arthropod Borne Dis..

