# Supplementary material for: Synergistic Control of Blattella germanica by Beta-Cypermethrin and Metarhizium anisopliae: Disruption of Gut Microbiota, Histopathology, and Detoxification Systems
Source: Insects. 2026 Aug 26;17(9):896. doi: 10.3390/insects17090896 (PMC13607507; doi:10.3390/insects17090896)
Supplement: Supplementary file 1 [file insects-17-00896-s001.zip › insects-4496202-supplementary.pdf]

Table S1. Effects of acetone on spore germination, colony diameter, and sporulation of *M. anisopliae*

Treament	Spore germination inhibition rate(%)*	Colony diameter inhibition rate(%)*			Number of produced conidia inhibition rate(%)*
		5 d	10 d	15 d	
0.5% acetone	1.45±0.51a	1.25±0.72a	2.37±1.02b	3.44±0.22a	32.53±3.31a
1% acetone	1.71±2.60a	0.00±2.89a	5.33±1.37b	4.96±0.22a	24.21±1.07a
5% acetone	4.01±0.31a	23.75±6.50b	26.04±9.22c	26.72±10.14b	80.59±6.94b
10% acetone	10.28±0.81b	61.25±2.17c	64.40±2.73d	52.29±2.42c	99.21±0.07c

*Different lowercase letters represent significant differences between groups ($P<0.05$)

Table S2. Compatibility index analysis of acetone and beta-cypermethrin with *M. anisopliae*

Treament			BI	CC
acetone	CYP(μg/mL)	Ma(cfu/mL)		
0.5%	0	0	84.25	C
1%	0	0	87.07	C
5%	0	0	52.39	MT
10%	0	0	31.74	T
1%	0	1×10^7	91.17	C
	0	1×10^8	87.10	C
	0	1×10^9	86.55	C
0	1	1×10^7	89.23	C
0		1×10^8	81.69	C
0		1×10^9	80.33	C
0	5	1×10^7	85.03	C
0		1×10^8	78.19	C
0		1×10^9	73.24	C
0	10	1×10^7	82.67	C
0		1×10^8	74.81	C

0	1×10^9	71.82	C

Note: BI: Biological Index; CC: Compatibility Classification; C: Compatible MT: Moderately Toxic; T: Toxic.The BI value indicates the level of compatibility, where a BI value of 0 to 41 is toxic, 42 to 66 is moderately toxic, and greater than 66 is compatible.

Table S3.Operational taxonomic unit (OTU) numbers and alpha diversity indices of gut microbiota for each sample

Sample ID	OTUs	ACE	Chao1	Simpson	Shannon	Coverage
C	384±2.33a	391.35±1.76a	395.30±0.62a	0.02±0.00a	4.63±0.07a	0.999
Ma	383±2.03a	392.21±1.79a	392.74±2.02a	0.02±0.00a	4.61±0.08ab	0.999
CYP	371±1.20b	386.46±2.30a	392.93±2.50a	0.02±0.00a	4.58±0.05ab	0.998
CYPMa	374±4.67b	388.70±4.94a	389.90±8.28a	0.35±0.00b	4.38±0.06b	0.999

C: control group; M: M. anisopliae group; CYP: beta-cypermethrin group; CYP_Ma: M. anisopliae and beta-cypermethrin combination group; different lowercase letters represent significant differences between groups (*P*<0.05)
